# The role of internal variability and external forcing on southwestern Australian rainfall: prospects for very wet or dry years

**DOI:** 10.1038/s41598-023-48877-w

**Published:** 2023-12-07

**Authors:** Surendra P. Rauniyar, Pandora Hope, Scott B. Power, Michael Grose, David Jones

**Affiliations:** 1 Australian Bureau of Meteorology, Melbourne, Australia; 2 ARC Centre of Excellence for Climate Extremes, Sydney, Australia; 3 School of Earth, Atmosphere and Environment, Monash University, Melbourne, Australia; 4 Centre for Applied Climate Sciences, University of Southern Queensland, Toowoomba, QLD Australia; 5 CSIRO Oceans and Atmosphere, Hobart, Australia

**Keywords:** Climate sciences, Climate change, Attribution, Climate-change impacts, Climate-change mitigation, Projection and prediction

## Abstract

The cool-season (May to October) rainfall decline in southwestern Australia deepened during 2001–2020 to become 20.5% less than the 1901–1960 reference period average, with a complete absence of very wet years (i.e., rainfall > 90th percentile). CMIP5 and CMIP6 climate model simulations suggest that approximately 43% of the observed multi-decadal decline was externally-forced. However, the observed 20-year rainfall anomaly in 2001–2020 is outside the range of both preindustrial control and historical simulations of almost all climate models used in this study. This, and the fact that the models generally appear to simulate realistic levels of decadal variability, suggests that 43% might be an underestimate. A large ensemble from one model exhibits drying similar to the observations in 10% of simulations and suggests that the external forcing contribution is indeed larger (66%). The majority of models project further drying over the twenty-first century, even under strong cuts to greenhouse gas emissions. Under the two warmest scenarios, over 70% of the late twenty-first century years are projected to be drier than the driest year simulated during the 1901–1960 period. Our results suggest that few, if any, very wet years will occur during 2023–2100, even if strong cuts to global emissions are made.

## Introduction

The strong spatial gradient in rainfall in southwest Western Australia (SWWA) is evident on the ground: from the tall trees in the far southwest through the wheat belt and into the dry interior, over a distance of approximately 500 km^1^. Thus, any small shift in the weather patterns north or south can strongly influence rainfall totals across the region, with consequences for dryland farming, ecosystems, and regional water supplies^2^. SWWA has very dry summers and wet winters indicating a region highly influenced by the seasonal cycle of weather systems. Rainfall in the cool season (May to October) contributes about 70% of the annual total, so the May to October period is the main focus of this study. Cool season rainfall comes primarily from the fronts and lows that cross the region from the west^3, 4^, while warm season rainfall is linked to the west coast trough, thunderstorms or the break-down of tropical lows and cyclones typically moving down from the tropics^5^.

**Figure 1:**
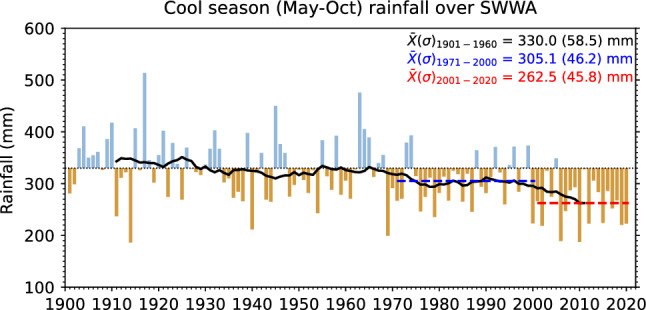
Time-series of area-averaged observed May to October rainfall 1900–2020 averaged over the South West Coast drainage division (see map in Fig. 2) using 1.5° × 1.5° regridded Australian Gridded Climate Data (AGCD) monthly rainfall data. Blue and brown bars represent rainfall above and below the average of the 1901–1960 reference period (horizontal black dotted line), respectively. The black solid line represents 20-year running average rainfall. Blue and red horizontal dashed lines represent the 1971–2000 and 2001–2020 averages, respectively. The average values for these periods are shown in the top-right corner, with the corresponding periods' standard deviation shown inside the parentheses.

Cool season rainfall in southwest of Australia is strongly influenced by mid-latitude weather systems, while climate modes including the El Niño Southern Oscillation (ENSO) have minimal impact^6^. Cool season rainfall (Fig. 1) prior to the 1960s exhibited interannual variability, with some years experiencing well above average rainfall, while some of the other years experienced well below average rainfall. After the 1960s, however, those very wet seasons disappeared, and the number of troughs and rainfall from those weather systems both declined^7–11^. A further downward step in decadal rainfall in SWWA occurred in the late 1990s^12, 13^. This coincided with a downward step in other regions around the world^14–16^. A decline in SWWA cool season rainfall is evident in almost all CMIP5 climate models under all historical forcings^17^.

By the early 1990s, Nicholls and Lavery^18^ presented a discussion of a potential role for human-caused climate change. Since the 1990s, multiple generations of climate models and scientific assessments have supported a role for human influence^19–21^ and ongoing drying^22–24^, with the IPCC Sixth Assessment Report showing that further future drying is *very likely* in SWWA^25^. While previous research examined rainfall changes under the higher emissions scenarios, the current outlook for global emissions suggests that we may avoid the very highest scenarios. And while current pledges are more consistent with mid-range scenarios that tend to exhibit 2 to 3 °C global warming^26–28^, there is a great deal of effort around the world now to restrict global temperature to "well below 2 °C above preindustrial levels" and pursue efforts "to limit the temperature increase to 1.5 °C above preindustrial levels" (Paris agreement^29^). The temperature change evident in the lowest scenarios used in CMIP5 and CMIP6 are more consistent with the Paris agreement. So we also examine rainfall change under these lower-emission pathways too.

In this study, we analysed observations and output from CMIP5 and CMIP6 models using new approaches^30^ to: (i) estimate the relative roles of external forcing and internal variability in 2001–2020 cool-season rainfall decline, (ii) explore the time of emergence of the climate change signal in SWWA rainfall, and (iii) estimate multi-decadal rainfall change over coming decades with and without emission mitigation efforts. In addition, we examined a 40 member large-ensemble of the ACCESS-ESM1.5 climate model^31^ under different forcing conditions (i.e., preindustrial, historical and future—under different emissions pathways) to estimate the likely change in the frequency of very wet (and dry) years in coming years and decades, including the influence from natural variability. We define a year as very wet (dry) when rainfall in that year exceeds (remains below) the 90th (10th) percentile value of the 1901–1960 period. ACCESS-ESM1.5 was chosen for special attention since it represents one of the driest projections in CMIP5 or CMIP6^32^.

The rest of the paper is organized as follows. “Results” section provides the findings based on the analysis of observations and climate models. “Discussion” section summarizes the key findings of this study, discusses possible explanations for the observed decline and suggests future work. “Methods” section includes a brief description of data, climate models and methods used in this study, including the model evaluation results.

## Results

### How unusual was the 2001–2020 low rainfall period compared to the full record?

**Figure 2:**
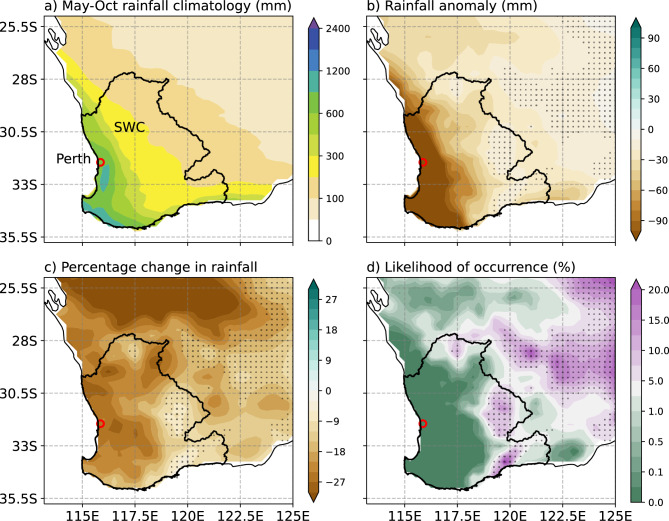
Spatial distribution of observed cool season (May–October) rainfall and associated statistics. (**a**) rainfall climatology for the 1901–1960 period, (**b**) relative anomaly and (**c**) percentage change in rainfall for the 2001–2020 period relative to climatology shown in (**a**), and (**d**) the likelihood of the 2001–2020 period observed change arising from random variability computed using the bootstrapping method described in “Methods”. The region considered in this paper is the South West Coast drainage division shown inside the black polygon. The city of Perth is marked with a red open circle. Stippling indicates the regions where the rainfall change is not statistically significant at the 5% level.

**Figure 3:**
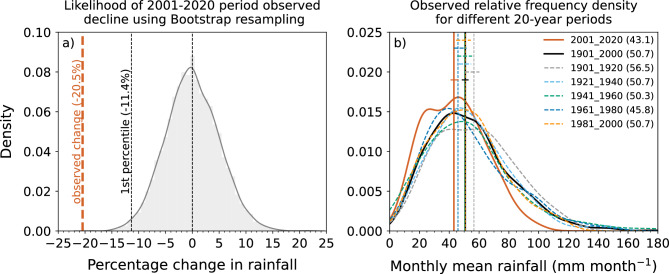
(**a**) Relative frequency density (RFD) of % change in rainfall between the recent 20-year period average and the first 60-year period average, calculated by random re-sampling of individual years (with replacement) from the full historical period (120 years; 1901–2020) 10,000 times and computing the % difference each time. Black (thin) dashed vertical lines in (**a**) from left to right represent the 1st percentile and median of the observed resampled distribution. The observed % change for the 2001–2020 period relative to the mean of 1901–1960 is indicated with the heavy dashed vertical line in orange. This line (at -20.5%) indicates that the observed change is outside the bootstrapped distribution. (**b**) RFDs of area-averaged monthly rainfall through the cool season (May to October) for six different 20-year blocks from 1900–1919 through to 2001–2020 period shown as coloured lines (see legend in (**b**)). The RFD for the full early period (1900–2000) is also shown for comparison (solid black line). The vertical lines represent the median values of the distributions. The median values (mm month^−1^) are shown in the parentheses in the legends. The horizontal segments indicate the 5th and 95th percentile confidence interval of the medians for each period, estimated using bootstrapping of monthly average rainfall for the period shown.

This section examines the observed characteristics of the SWWA rainfall (Fig. 2). The spatial pattern of the climatological May to October rainfall shows that the greatest totals occur along the coastal margins (Fig. 2a). The relative difference in rainfall between 2001–2020 and 1901–1960 is large in absolute terms (Fig. 2b: greater than 90 mm in the west) and as a percentage (Fig. 2c: with many areas seeing a reduction of around 20%). For regions that have experienced the largest declines, according to our analysis (see “Methods” section), it is exceptionally unlikely (likelihood < 0.001) that a decline of the magnitude experienced could occur from natural internal variability only (Fig. 2d). The severity of the situation can be seen more clearly in Fig. 3a which shows the observed percentage change of area-averaged cool season rainfall for the 2001–2020 period from the 1901–1960 period average (marked by the heavy dashed vertical coloured line) is outside the range of all possible differences between the last 20-year period and the first 60-year period from the observed record resampled 10,000 times. Examining the temporal variability, each month from the cool season (May to October) in the most recent 20 years (2001–2020) was likely to have lower rainfall than months from any other 20-year period of the twentieth century (Fig. 3b). All other 20-year periods had some months with more than 120 mm of rainfall, but none were evident in the 2001–2020 period. Viewed in the context of past published work and analyses here, the extremely low rainfall totals in recent decades are part of an ongoing long-term downward trend in rainfall in SWWA.

### Contribution of external forcing to the observed SWWA rainfall decline during 2001–2020 relative to 1901–1960

As evident from analysing the observations, the 2001–2020 decline in rainfall was exceptional and cannot be replicated through resampling of interannual variability demonstrated in the first half of last century. Climate models provide a tool to explore the possible range of internal variability and compare that with the anthropogenic forced signal. In this section, we estimate the relative contributions of external forcing and internal variability to the observed decline by using the first ensemble member of CMIP5 (r1i1p1) and CMIP6 (r1i1p1f1) model simulations (Supplementary Tables S1 and S2) and also using large ensembles from two CMIP6 climate models and the results are described below.

### Using CMIP5 and CMIP6 multi-model ensemble

**Figure 4:**
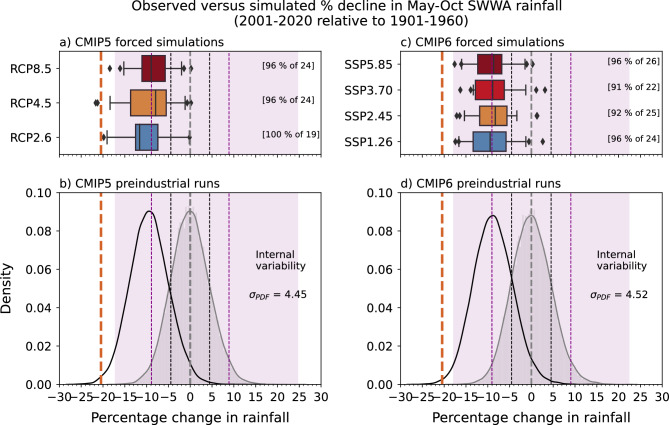
Statistics relating to the observed changes in CMIP5 (left) and CMIP6 (right) models. The boxplots represent the spread of percentage change from the first ensemble member of CMIP5 (**a**) and CMIP6 (**c**) under the scenarios shown. Multi-model median (MMM; line in box), inter-quartile range (IQR; box), whiskers (5th and 95th percentiles) and circles (outliers) shown. The number of models under each scenario and the percentage of models with the same sign as the MMM is shown inside the square brackets for (**a**) CMIP5 and (**c**) CMIP6. Lower panels: density functions of percentage changes from CMIP5 (**b**) and CMIP6 (**d**) preindustrial control simulations. Pink shading represents the full range, and the dashed lines in purple and black colours mark the two and one standard deviations, respectively. The black bell curves in (**b**) and (**d**) represent the internal variability in the present climate and were obtained by shifting the shaded grey curves in (**b**) and (**d**) to their left by the magnitude of externally-forced response (i.e., MMM). Observed decline of 20.5% is also marked as a red dashed vertical line.

To further examine the unusualness of the observed and model-simulated 2001–2020 drying, we compare the observed anomaly against the possible range of multi-decadal rainfall changes that can arise due to internal variability in the global climate models as seen in their preindustrial control simulations (Fig. 4). The distribution shown in the bottom panel of Fig. 4 represents the range of possible 20-year percentage change relative to any 60-year average due to internal variability alone (see “Methods”). The observed percentage change in 2001–2020 compared to 1901–1960, marked in Fig. 4 at less than -20%, is much lower than, and outside the modelled range, of all possible 20-year rainfall anomalies due to internal variability across the models.

According to the models, a modelled decline of the same magnitude as observed is exceptionally unlikely without external forcing and is consistent with our earlier results based on random resampling of observations (Fig. 2a). Although, even including all historical forcing runs, only a few models in CMIP5 (ACCESS1.3, GFDL-ESM2G and MPI-ESM-LR) and none in the CMIP6 ensemble simulate a rainfall decline as large or larger than the observed 20-year decline. To estimate the externally-forced change, we computed the average of the multi-model median (MMM) change across each scenario—which will average out and remove internal variability. This gives a decline of 9.3% in CMIP5 and 8.8% in CMIP6 (9.1% mean). Comparing the all-forcing change (average of CMIP5 and CMIP6) to the observed (−20.5%), we might simply conclude that 44% of the observed decline is due to external forcing (see Supplementary Tables S3 and S4 for more details). Including more models to heighten the confidence in the signal reveals a similar value of 43% (i.e., including those models with a shorter preindustrial period—200 years—now 40 models in CMIP5 + RCP8.5 and 33 in CMIP6 + SSP5.85).

The observed rainfall decline is well beyond the range of internal variability simulated by most CMIP5 and CMIP6 climate model runs analysed (Fig. 4). However, the observed rainfall for the 2001–2020 period will reflect a combination of natural variability and the forced (anthropogenic) response approximated by the CMIP ensemble. Statistical theory indicates that for normally distributed variables, 95% of values lie within ± two standard deviations of the mean. In our case, the standard deviation of the internal variability is equivalent to 4.5% of mean, so the range is equal to ± 9.0% of the mean. Adding this 9.0% to the 9.1% externally-forced decline (from above) gives a total of 18.1%, which is still smaller than the observed reduction for 2001–2020 of 20.5%. However, the observed change is now encompassed within the modelled range in present climate (i.e., black bell curve in Fig. 4), but the likelihood is still low (i.e., 0.4% and 0.55% according to CMIP5 and CMIP6 models, respectively). There may also be processes that the models are missing or misrepresenting^30, 33, 34^.

The fact that the observed rainfall decline is qualitatively similar to the model projections (significant and ongoing) but about twice as large raises some significant science and policy questions. One interpretation is that SWWA has, by chance, sampled extreme natural variability that strongly reduces rainfall when the anthropogenic signal is also at its largest. Another interpretation is that climate models are underestimating the rainfall response to anthropogenic forcing, and perhaps the future will see even larger declines again. We know from the results presented above that natural variability acting together with the external forcing can cause drying as large as observed, but this only occurred in 0.4–0.55% of cases. This very low likelihood suggests that externally-forced drying in the models is too weak and/or there was an extraordinarily large internally generated drying event in the real world.

### Using large ensembles from single models

An alternative estimate of the contribution of external forcing to the observed decline can be obtained from large ensembles of individual models. The signal and effect of variability across a large ensemble from a single model can reveal other facets as compared to a multi-model ensemble as uncertainty in the internal variability due to inter-model variability caused by differing physics, dynamical cores, and resolutions can be eliminated. A 40-member ensemble from ACCESS-ESM1.5 has an ensemble mean reduction of 13.5% rainfall in 2001–2020 relative to 1901–1960, suggesting 66% of the observed decline was externally forced. We also see that 10% of 160 simulations in ACCESS-ESM1.5 (i.e., 40-member for each SSP) match the observed rainfall decline between 1901–1960 and 2001–2020. The ACCESS-EMS1.5 ensemble was generated from different initial conditions by using different branching points from the preindustrial control simulations at 20-year intervals^31^. Similarly, analysis of a 50-member ensemble from CanESM5^35^ suggests that external forcing contributed about 54% of the observed decline. However, all the CanESM5 ensemble members (170 in total; 50-member for each SSP except for SSP126 which has 20 ensemble members) underestimated the magnitude of the observed decline (not shown).

ACCESS-ESM1.5 has a strong historical and projected drying signal relative to other CMIP6 models, so if we assume this model has a reliable forced response, this suggests that human influence played a major role in the rainfall decline, and if internal variability is accounted for, there was a heightened chance of experiencing a decline as large as observed.

### Emergence of the externally-forced signal and what does that mean for the future?

In this section, we estimate the date at which the externally-forced signal in modelled 20-year SWWA cool season rainfall emerges from its preindustrial variability and what that means for the future given that external forcing played a large role in the 2001–2020 decline over SWWA as we showed above. We define that the signal emerges from the noise when these three conditions are met, following Rauniyar and Power^30^: (i) the MMM value of 20-year block goes beyond one standard deviation of preindustrial (internal) multidecadal variability, (ii) the MMM stays away from that envelop for the rest of the simulations out to 2100 and (iii) at least 75% of the models show the same sign of change as the MMM.

**Figure 5:**
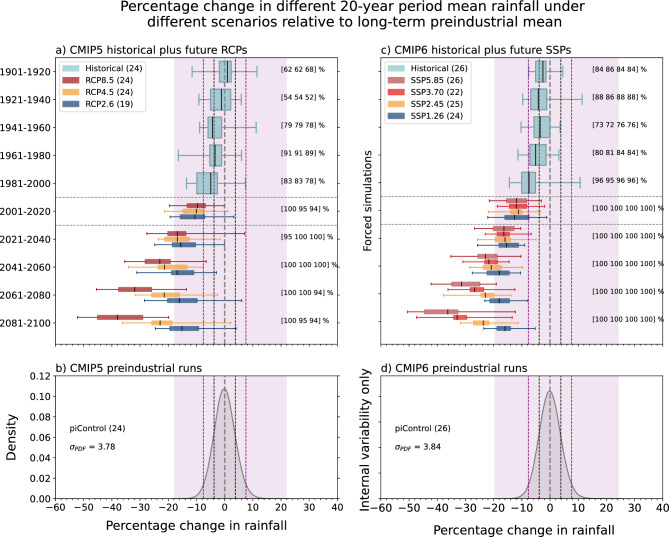
Assessing 20-year average percentage changes in southwestern Australian cool season rainfall in the CMIP5 (left) and CMIP6 (right) climate models against the distribution of internal variability. Changes are relative to each model's long preindustrial control average and the spread among the models are shown as boxplots for different 20-year blocks from 1901–1920 through to 2081–2100. The density functions shown in the lower panel represent the range of percentage change in 20-year period due to the internal variability alone (see “Methods”). Purple and black vertical dashed lines indicate two and one standard deviation, respectively and the pink shading represent the full range of the internal variability. Number of models used in each scenario is shown in the parenthesis in the legends. Percentage of models with the same sign as in multi-model median (MMM) are shown in square brackets on the right side of the top panel in the order of high (left most) to low emission (right) scenarios.

Figure 5 shows the modelled unforced 20-year variability (compared to 500-year-long preindustrial mean) in SWWA rainfall ranges from − 18 to + 22% in CMIP5 and with a slightly wider range from − 19 to 24% in CMIP6. Assuming a gaussian distribution, the one standard deviation is equivalent to 3.78% in CMIP5 and 3.84% in CMIP6, and we might expect that the magnitude of a 20-year change could be up to ± two standard deviations (~ 8%) beyond any externally-forced signal (MMM) 95% of the time.

Figure 5 also shows that prior to 1980 the climate change signal is not that clear—the 20-year MMM value is well within the bounds of the unforced interdecadal variability of the models and there is variability from decade to decade. But after 1981–2020 the MMM sits outside one standard deviation of the multi-model preindustrial decadal variability, more than 75% of the models agree on a drying signal and after this time there is a consistent decrease in decadally-averaged rainfall. This suggests that externally-forced drying became dominant in the 1981–2020 period, and this period can be defined as the time of emergence for cool-season rainfall in the SWWA region. In the more recent period (2001–2020), the signal of global warming is very clear, as the MMM 20-year averages are more than two standard deviations drier than the multi-model preindustrial decadal variability, and only one model is wetter than the preindustrial mean.

In the near term (2021–2040) all scenarios show a similar change. The projected rainfall decrease grows to more than four model-estimated standard deviations, but it is only with further declines into the next 20-year period that the MMM matches the observed 2001–2020 decline. The strong modelled rainfall reductions in the near future suggest that a dry near-future compared to preindustrial climate is extremely likely, and conditions could sit well outside preindustrial variability, but there is some possibility that it won't be as dry as 2001–2020 if internal variability pushes towards wet conditions. After the mid-century, differences between responses evident under various emissions are much clearer. This can be clearly seen from 2041 to 2060 as the impact of greenhouse gas reductions starts to become apparent under lower emissions scenarios, so strong mitigation of emissions act to limit the decline.

If we follow a high emission pathway (RCP8.5 or SSP5.85), by the end of the century all but one model projects that 2081–2100 will be drier than any 20-year period in the preindustrial simulations with the magnitude of MMM drying being less than 35% of long-term preindustrial mean (Fig. 5). Furthermore, 98% of models (49 out of 50) project that 2081–2100 will be drier than the observed rainfall anomaly in 2001–2020. However, strong mitigation of emissions limits this decline, and some recovery of decadal rainfall is evident by the end of the century under low emissions scenarios. But even under the lowest emission scenarios (RCP2.6 and SSP1.26) the MMM of 20-year rainfall at the end of the century is 15% below the preindustrial average which is the 0.005% percentile of the preindustrial distribution.

Supplementary Tables S3 and S4 shows that the MMM projected rainfall changes are very similar in CMIP5 and CMIP6 for their low, medium, and high emissions scenarios (i.e., RCP2.6 vs SSP1.26, RCP4.5 vs SSP2.45 and RCP8.5 vs SSP5.85). Furthermore, the time evolution of models' 20-year rainfall change relative to their 1901–1960 mean (Supplementary Tables S3 and S4) shows that the magnitude of MMM drying will be larger than the observed decline in 1971–2000 even under the strong mitigation of emissions for the remainder of the twenty-first century. Note that future rainfall will be determined by both natural variability and external forcing. If the externally-forced signal is large enough, the relatively modest natural variability is simply overwhelmed by the human caused drying trend.

### Prospects of very wet and dry years in future

**Figure 6:**
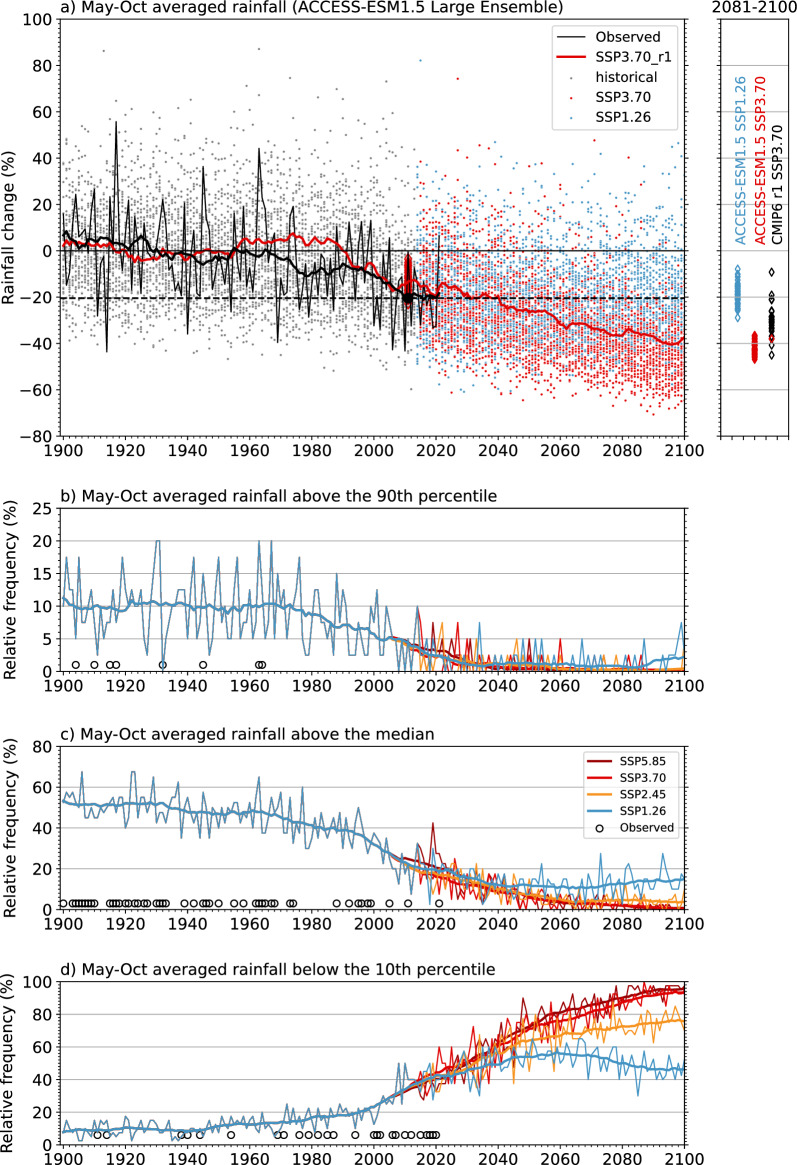
Prospects of very wet and dry years in SWWA. (**a**) SWWA May to October averaged rainfall relative to a 1901–1960 baseline for 40 realisations of ACCESS-ESM1.5 under historical forcing (grey circles) and under two different future emissions pathways (SSP3.70 in red and SSP1.26 in blue circles). Observed rainfall is shown in the black line, with the 20-year running mean in the thick black line. The modelled 20-year averaged time-series of the first ensemble member (r1) of ACCESS-ESM1.5 under historical plus SSP3-7.0 scenario is shown in a thick red line. The percentage change in the 2001–2020 average is shown as a thin red diamond for each ensemble member and a black-filled circle for the observations. Comparison of the spread in 2081–2100 rainfall change between 40 realisations of ACCESS-ESM1.5 under SSP1-2.6 (blue diamonds) and SSP3-7.0 (red diamonds) and the first realisation of CMIP6 models under SSP3-7.0 (black diamonds) is shown in the top right box. The lower panels are summary statistics of the plot above. The lines in panels (**b**) and (**c**) show the relative frequency of the ACCESS-ESM1.5 40 ensemble members that exceed the 90th percentile and median rainfall of their 1901–1960 period under historical and four different SSPs, as shown in the legend. Panel (**d**) shows the relative frequency of the ACCESS-ESM1.5 40 ensemble members for years that show rainfall below the 10th percentile of the 1901–1960 period. The smooth lines in panels b-d are 20-year running means. Open black circles in panels (**b**) and (**c**) indicate the years when observed May to October SWWA rainfall exceed the 90th percentile and median of 1901–1960 period records, while the same in panel (**d**) for the rainfall below the 10th percentile of 1901–1960 period.

An obvious signal in the observed record is the total absence of very wet May to October seasons in recent decades (i.e., season with rainfall > the 90th percentile of seasonal rainfall during 1901–1960). Even though the decadal climate is projected to remain dry, could very wet years still occur in the future? And does the answer depend on the degree to which net emissions are reduced? To address these questions, we use the 40-member ensemble of ACCESS-ESM1.5. As noted above, this model features a strong drying signal in the region, though it does not fully replicate the observed drying trend. Given 40 simulations, we can assess the probability of any decile including very wet years (top decile)—where 0/40 simulations for a given year is taken simply to indicate zero probability and 40/40 is taken to indicate 100% probability that the event will occur (Fig. 6).

Figure 6a shows, as expected, that the variability of individual years can be much larger than decadal variability. The ensemble range is large—in some years cool season rainfall in the ACCESS-ESM1.5 ensemble is more than 60% above the 1901–1960 average—higher than any year in the observed data. Very wet years (> 90%) still occur occasionally after 2033 (Fig. 6b), regardless of the scenarios. However, they become increasingly less frequent over time, dropping from 10% of ensemble member to close to zero (i.e., 0.25%) at the end of the century under the two warmest scenarios (SSP5.85 and SSP3.70). Even under a relatively low emissions scenario (SSP1.26), fewer than 1.2% of simulated years after 2020 (38 out of a possible 3200 years) are very wet. For the same period, the likelihood is extremely low (1 in 3200) for rainfall larger than the simulated historical wettest year on record under SSP1.26, but slightly higher (4 out of 6400 years) under the two warmest scenarios (Supplementary Fig. S1).

Even the chance of more moderate rainfall—above the median of 1901–1960—also drops dramatically from the late 1990s onwards, remaining below 20% through the remainder of the century (Fig. 6c). Towards the end of the century, the probability of rainfall above the median could reach below 1% under the two high emission scenarios. However, strong mitigation of emissions will reduce the impact and lead to some recovery. At the end of the century, there is a 15% chance that some years will experience above 1901–1960 average rainfall under the low emission scenarios.

ACCESS-ESM1.5 generally projects a lower average SWWA rainfall than many of the other CMIP6 models (Fig. 6a, top right box) and its first realisation (r1i1p1f1) has lower interannual variability than observed (Fig. 9); thus, any estimate of extreme years from the ACCESS-ESM1.5 might be less wet than other models. Nevertheless, this general projection of a dramatic reduction (but not elimination) in the probability of very wet or even previously average years is a credible projection given a drying climate.

In contrast, the likelihood of rainfall below the 10th percentile of 1901–1960 increases consistently from 10 to 20% until 2000, rising to 40% by 2020 (Fig. 6d). Towards the end of the century, the likelihood is projected to be above 90% under the two warmest scenarios. According to the ACCESS-ESM1.5 model, this situation is projected to improve significantly under the lowest emission scenario. However, even with a marked reduction in emissions, the SWWA rainfall will be below the 10th percentile of 1901–1960 for more than 45% of the late twenty-first century. Simulated years below the lowest on record in 1901–1960 follow a similar pattern—comparing the scenarios—if we follow the two highest emissions scenarios, 70% of the simulated years in the late twenty-first century (2081–2100) will have rainfall less than the driest year in the early twentieth century (1901–1960) (Supplementary Fig. S1). However, under the lowest emissions scenario, the situation is not as bad, and in the late twenty-first century (2081–2100) only 20% of the simulated years will have less rainfall than the driest year during 1901–1960.

## Discussion

The observed south-west Western Australia (SWWA) May–October averaged (cool season) 2001–2020 rainfall is the driest 20-year period on record, with the average over this period 20.5% below the 1901–1960 mean. Random resampling of the observations implies a near-zero probability that a decline of this magnitude could have occurred from internal variability alone. Both CMIP5 and CMIP6 climate models suggest that the externally-forced drying has already emerged over SWWA during the late twentieth century, and multi-model ensembles indicate that about half of the observed post-2000 decline can be attributed to external forcing. However, nearly all CMIP5 and CMIP6 simulations considered here underestimated the magnitude of the observed drying for the 2001–2020 period. An estimate of the contribution of external forcing to the observed drying in SWWA from a large ensemble of the ACCESS-ESM1.5 model, which exhibits a stronger drying signal, is 66%, and 10% of the ACCESS-ESM1.5 model simulations match the observed 2001–2020 rainfall decline. The inability of most models to fully replicate the large drying seen so far leads to two possible conclusions: the rainfall in this region is more sensitive to greenhouse gas concentrations than is currently modelled; or factors other than climate change have coincidentally reduced rainfall during the recent period of anthropogenic climate change.

Into the future, regardless of the emissions scenario, both CMIP5 and CMIP6 climate models exhibit a forced signal that remains outside the range of internal variability in the preindustrial climate at the end of the century. But strong mitigation of emissions will reduce the rainfall changes from 65% of the 1901–1960 average under a business-as-usual scenario (RCP8.5 or SSP5.85) to 85% under a scenario with major emissions reductions (RCP2.6 or SSP1.26) suggesting future rainfall is highly dependent on the future trajectory of greenhouse gas emissions. At the end of the century, again according to the ACCESS-ESM1.5 model, some years with average rainfall will begin to return under the low emission scenarios. But very wet years are projected to be extremely rare towards the end of century under the top three emissions scenarios (i.e., 4 out of 2400 simulated years or 1 in 600), however a strong cut in emissions increases the likelihood substantially (i.e., 18 out of 800 simulated years or 1 in 45). In contrast, SWWA rainfall is projected to remain below the decile 1 value of 1901–1960 for approximately 45% of time during the late twenty-first century, even under a scenario with a marked reduction in greenhouse gas emissions (i.e., low emissions scenarios RCP2.6/SSP1.26). The projected decline in wet years as well as the climatological average suggests that even agriculture and other activities that can opportunistically use occasional wet years may well struggle, especially under higher levels of climate change.

Our new analysis and results emerging from the CMIP6 ensemble of models provide further clarity and nuance to earlier findings around the decline of rainfall in SWWA in the historical period^20^ and projections^23, 24^. The observed rainfall decline is better captured by the models compared to earlier single-model studies^21^, possibly due to the method to calculate the model areal-average rainfall that is less strict about the coastal boundary^36^, as described in the “Methods” section. The pattern of average CMIP6 rainfall projections is similar to those from CMIP5^25^, but the CMIP6 change is greater over the land than in CMIP5. We show that for the lower emissions scenario, the response in CMIP6 is also more emphatic than CMIP5. The large ACCESS-ESM1.5 ensemble highlights that a model's internal variability will produce decadal variability on top of the forced signal, and these nuances are worth exploring further as more results become available.

The observed record is only one realisation of the climate response to external forcing and we have shown that more than half of the observed post-2000 decline may be due to internal variability, thus the last two decades could have had a different rainfall signature. To explore that, large ensembles might help us better understand the potential range, particularly if combined with further evaluation of the circulation changes driving the rainfall change in both observations and models^37^. The circulation changes in projections^17^ will reflect the modelled mechanisms by which increasing greenhouse gases are altering the atmospheric circulation, but further research into how the circulation has changed in historical single forcing runs (e.g., aerosols only) could form a further study.

In addition to climate forcing, land surface changes associated with a shift from forestry to agriculture have been postulated to contribute to a reduction of how much rainfall is drawn from any passing weather system during the cool season in SWWA^38, 39^. Land cover changes^40^ are included in some of the CMIP6 histALL simulations (e.g., ACCESS-ESM1.5), but not others (e.g., ACCESS-CM2) however it is hard to pinpoint any impact without single forcing simulations from the same model. Results from Land Use MIP could form the basis of further study as they become available. Factors such as the Antarctic stratospheric ozone hole recovery and its impact are also worth further exploration, as we learn more about links to surface climate^41, 42^.

The rainfall reduction appears to stabilise in some models if we follow a very low emissions pathway and reach net zero emissions, indicated by change under RCP2.6 and SSP1-2.6 (Figs. 5 and 6). This might suggest that SWWA could return towards its 1900s climate during the twenty-second century after global atmospheric greenhouse gases stabilise^43^. However, this result is not strong and is likely to be model dependent. Using estimates of the climate at these latitudes after it stabilises after emissions stop increasing, we do not see a return to the very wet conditions evident in other periods with high levels of carbon dioxide such as in the Pliocene or under extended simulations into the twenty-second century^43–45^.

Palaeoclimate reconstructions of the recent era can also help assess the potential rainfall variability in an unforced world to help understand the potential variability we might see around the forced signal in coming decades. Reconstructions from tree rings and caves suggest that decadal rainfall variability in SWWA is significant, but primarily stochastic, and only weakly correlated with large-scale drivers such as ENSO, the Southern Annular Mode or local sea-surface temperatures^46^. From a site in the wheat belt there is evidence that multi-decadal dry spells have occurred before, and that the early 1900s were unusually wet in that region, suggesting that internal climate variability is an important factor in understanding decadal rainfall changes in SWWA^12^. Isotopes in cave structures and groundwater also highlight different aspects of the rainfall changes including variability in the most intense events of the year^47^ and groundwater variability. In the very wet south-west coastal region the recent decades have seen the lowest soil moisture in at least the last 800 years^48^, shallow karst aquifers are becoming disconnected from rainfall, and groundwater is failing to recharge.

In south-west Australia two desalination plants have helped supply water to the city of Perth and the Water Corporation has planned well for the extended dry informed by climate science. Away from the city and coast, regional towns and agriculture are dependent on surface water and increasingly also on groundwater. A warming and drying climate presents enhanced stress on plants across the region^49^ and adaptive measures in agriculture have already been applied such as changing planting to perennials, which also help with soil salinity (e.g., South Coast Natural Resource Management Inc 2018). There is high confidence in further rainfall declines in SWWA, and so there may continue to be demand for a transition to industries less reliant on water. However, strong mitigation could help reduce the extreme 20-year rainfall declines projected by models, and wet years may occur sporadically.

## Methods

### Data

Rainfall data is from the 5 km × 5 km Australian Gridded Climate Data (AGCD^50^), which is regridded to 1.5° × 1.5° using a conservative interpolation method^51, 52^ to match the coarse-resolution of CMIP models. Other observational datasets were also compared with AGCD: AWAP^53^, SILO^54^ and CRU TS4.05^55^. The distributions of the differences between these datasets were largely centred around zero (not shown), suggesting that each dataset would be a reasonable choice for this area. The few large differences were during very wet months (up to 20 mm) and likely linked to the different treatment of the background climatology.

The climate model data are from the Coupled Model Intercomparison Project Phase 5 (CMIP5^56^) and Phase 6 (CMIP6^57, 58^) including: preindustrial control; simulations forced with full historical forcing and the projections under different future emissions scenarios, three Representative Concentration Pathways (RCPs^59^) under CMIP5 (RCP2.6, RCP4.5 and RCP8.5) and four Shared Socio-economic Pathways under CMIP6 (SSP1-2.6, SSP2-4.5, SSP3-7.0, SSP5-8.5)—RCP2.6 and SSP1-2.6 representing a low emissions future and RCP8.5 and SSP5-8.5 a high emissions future. Note that the second number in the SSPs refers to the radiative forcing at 2100 in Wm^−2^, as in the RCPs, thus although the pathways differ, RCP8.5 and SSP5-8.5 can be considered high emissions scenarios, while RCP2.6 and SSP1-2.6 are both very low. Unless otherwise stated, we only use those models that provide at least 500 years of rainfall under preindustrial conditions to ensure a good estimate of the variability in pre-anthropogenic climate^19, 30^. Historical simulations (CMIP5: 1900–2005, CMIP6: 1900–2014) include all-forcings (histALL) including volcanic eruptions, solar variability, atmospheric greenhouse gas, aerosol, and ozone changes. See Supplementary Tables S1 and S2 for a full list of the models and available simulations. Models' data are also interpolated from their native spatial resolutions to a common 1.5° × 1.5° grid resolution using a conservative interpolation method^51, 52^ prior to computing the area-averaged rainfall time-series over the South West Coast drainage region (Fig. 2).

### Methodology

We performed Bootstrap resampling^60^ to estimate the likelihood of occurrence of the recent rainfall decline by random chance. This is done by random resampling of individual years (with replacement) of May–October average full historical period rainfall (120 years: 1901–2020) 10,000 times, with an assumption of zero persistence. This is justified as no statistically significant autocorrelation is found in the area-averaged detrended cool season rainfall (not shown). Then the percentage change for the last 20-year average rainfall (i.e., 2001–2020 average) compared to the first sixty years average rainfall (i.e., 1901–1960 average) is computed for each resample. This supplied 10,000 values which are used to estimate the probability density function (PDF) against which the actual decline is compared and the probability of occurrence due to its own statistical variability is determined.

**Figure 7:**
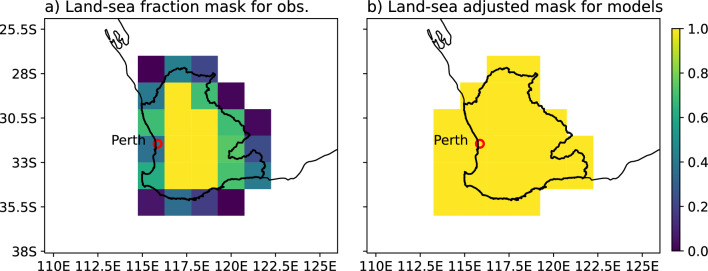
The 1.5 degree grids (**a**) land-sea fraction mask (LSFmask) used in the observations, where 1 mark fully land and zero marks fully ocean and (**b**) land-sea adjusted mask (LSAmask) used in the CMIP data to capture the bias of off-shore rainfall.

To capture the rainfall signal over the small SWWA region in the climate models the full grid points were included in the spatial average (Fig. 7). They include some sub-grid points defined as ocean, however this approximation better captures the rainfall signal as there is a known climate model bias whereby rainfall is modelled off-shore but observations suggest that it should actually have fallen over land^36^.

### Evaluation of climate models

**Figure 8:**
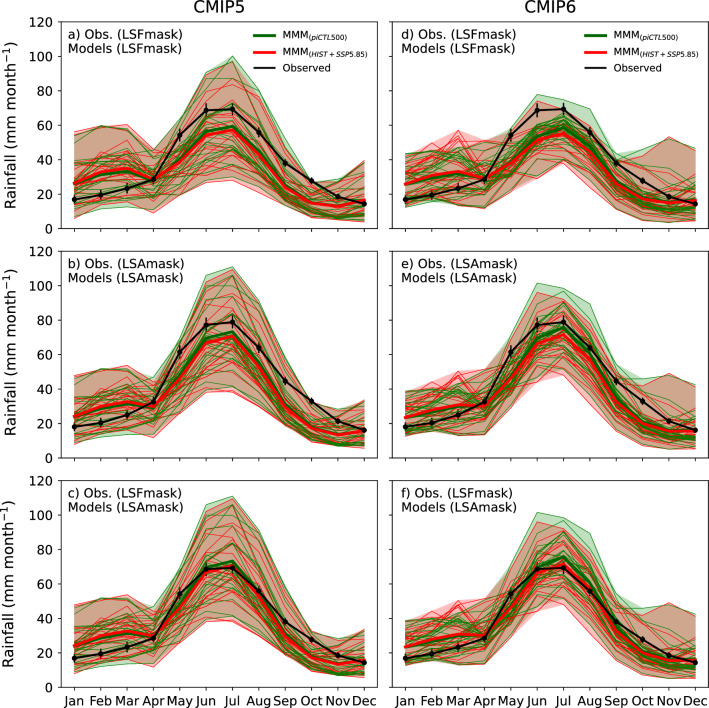
The seasonal cycle of SWWA area-averaged rainfall in the AGCD observations (black), the piControl (green) and the histALL (red) with the multi-model mean in bold. Each row shows which masks are applied to compute the area-average rainfall and are shown in the panels heading in parenthesis after the obs. and models, where 'LSFmask' and 'LSAmask' represent the masks shown in Fig. 7a and b, respectively. Vertical error bars on the monthly average AGCD rainfall represent the 5th and 95th percentiles confidence interval estimated using bootstrapping of monthly average rainfall. Left panels are CMIP5 and right are CMIP6.

The strong seasonal cycle in SWWA rainfall is a good index of whether the model processes are adequately representing the weather systems of the region and the shape and magnitude of each model's seasonal cycle was evaluated (Fig. 8). The AGCD average is shown in black and the preindustrial in green and histALL in red. The range of each is shaded and the multi-model mean is in bold. The comparison of the result averaged over different regions is also included in the top row compared to the next. Using the wider region (middle row) improves the seasonal cycle of the multi-model mean. All models from both CMIP5 (left column) and CMIP6 (right column) represent the pattern of the seasonal cycle well and are included.

**Figure 9:**
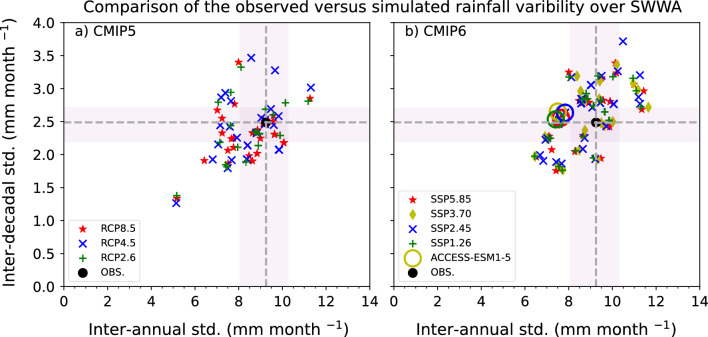
Comparison between the observed and model-simulated SWWA cool-season (May to October) rainfall standard deviations (mm month^−1^) for the period 1901–2020. Inter-annual and decadal standard deviations are shown along the X-axis and Y-axis, respectively. The black-filled circle represents the observations and the light gray shading represents the 5% to 95% confidence interval of the standard deviation (obtained using bootstrapping). The histALL simulations extended with the RCP8.5/SSP5.85, RCP4.5/SSP2.45, and RCP2.6/SSP1.26 scenarios are respectively shown using red stars, blue crosses and green plus symbols, and the SSP3.70 results are indicated by the yellow diamonds. CMIP5 results are on the left and CMIP6 on the right. ACCESS-ESM1.5 is marked with a circle.

We also assess how well the models simulate year-to-year and decade-to-decade variability of SWWA rainfall in their historical simulations, extended with each RCP or SSP to 2020 (Fig. 9). Many of the CMIP6 models have similar decadal variability to observations but almost all have reduced year-to-year variability; the majority have reduced variability on both timescales. The CMIP5 model results are more spread, and many sit outside the range of observed variability, particularly in the quadrant showing reduced variability, but some models have greater variability than observed, while several have higher decadal variability but reduced interannual variability.

We also compared the magnitude of the very wet year (i.e., rainfall above the 90^th^ percentile of the 1901–1960 records) in each ACCESS-ESM1.5 ensemble member against the observations. We found that the spread for very wet rainfall in the ACCESS-ESM1.5 across the 40 ensemble members is large and ranges from 18 to 36% above the 1901–1960 average but, encompasses the observed 90th percentile of 22%.

### Estimation of modelled internal variability

To estimate the probability density function (PDF) of the modelled internal variability, we used climate models with at least 500 years of simulations under preindustrial control simulations. For each preindustrial model, we randomly resampled the 500-years of data 1000 times and computed the percentage change of the last 20 year's average rainfall compared to the average of any non-overlapping six decades. This results in a sample of 24,000 values (24 models times 1000) of percentage rainfall changes for CMIP5 and 25,000 values for CMIP6 (25 models times 1000), which is then used to estimate the relative frequency of 20-year average rainfall changes relative to any non-overlapping six decades due to internal climate variability. The one and two standard deviations of this distribution are shown in Fig. 4. This process is repeated, however this time the percentage changes in rainfall in 20-year blocks in 500-years of pre-industrial simulations are computed relative to the long-term pre-industrial mean for each time and for each model. Finally, those values are used to estimate the PDF of the possible range of 20-year change that can arise due to internal climate variability only. This PDF and its associated statistics are used to estimate the timing of when the signal due to external forcing emerges from the internal variability and estimate the likelihood of future rainfall changes under different emission scenarios (Fig. 5).

### Supplementary Information

Supplementary Information.

## Data Availability

The datasets and codes used and/or generated in this study are stored in the National Computational Infrastructure (NCI) repository which is supported by the Australian Government. Relevant materials are available from the corresponding author on reasonable request. The CMIP5 and CMIP6 data used in this study is publicly available through the Program for Climate Model Diagnosis and Intercomparison (https://esgf-node.llnl.gov).

## References

[1] Fletcher AL, Chen C, Ota N, Lawes RA, Oliver YM (2020). Has historic climate change affected the spatial distribution of water-limited wheat yield across Western Australia?. Clim. Change.

[2] Power SB, Sadler B, Nicholls N (2005). The influence of climate science on water management in Western Australia. Bull. Am. Meteorol. Soc..

[3] Pepler AS (2020). The contributions of fronts, lows and thunderstorms to southern Australian rainfall. Clim. Dyn..

[4] Pook MJ, Risbey JS, McIntosh PC (2012). The synoptic climatology of cool-season rainfall in the central wheatbelt of Western Australia. Mon. Weather Rev..

[5] Pepler AS, Hope P (2018). Orography drives the semistationary West Australian summer trough. Geophys. Res. Lett..

[6] IOCI (2002). Indian Ocean Climate Initiative (IOCI): Climate Variability and Change in South West Western Australia.

[7] Wright PB (1974). Seasonal rainfall in southwestern Australia and the general circulation. Mon. Weather Rev..

[8] Bates BC, Hope P, Ryan B, Smith I, Charles S (2008). Key findings from the Indian ocean climate initiative and their impact on policy development in Australia. Clim. Change.

[9] Hope, P. *Projected Future Climate Changes in South-West Western Australia*. *BMRC Research Report no. 125, Bureau of Meteorology Research Centre* (2006).

[10] Raut BA, Jakob C, Reeder MJ (2014). Rainfall changes over Southwestern Australia and their relationship to the southern annular mode and ENSO. J. Clim..

[11] Pepler AS, Dowdy AJ, Hope P (2021). The differing role of weather systems in southern Australian rainfall between 1979–1996 and 1997–2015. Clim. Dyn..

[12] O’Donnell AJ, McCaw WL, Cook ER, Grierson PF (2021). Megadroughts and pluvials in southwest Australia: 1350–2017 CE. Clim. Dyn..

[13] Priestley SC (2023). Caves demonstrate decrease in rainfall recharge of southwest Australian groundwater is unprecedented for the last 800 years. Commun. Earth Environ..

[14] Verdon-Kidd DC, Kiem AS, Moran R (2014). Links between the big dry in Australia and hemispheric multi-decadal climate variability-implications for water resource management. Hydrol. Earth Syst. Sci..

[15] Jones RN, Ricketts HJ (2017). Reconciling the signal and noise of atmospheric warming on decadal timescales. Earth Syst. Dyn..

[16] Hope P, Ganter CJ, Jubb I, Holper P, Cai W (2010). Recent and projected rainfall trends in south-west Australia and the associated shifts in weather systems. Book of Proceedings from Greenhouse 2009 Conference.

[17] Seager R (2019). Climate variability and change of mediterranean-type climates. J. Clim..

[18] Nicholls N, Lavery B (1992). Australian rainfall trends during the twentieth century. Int. J. Climatol..

[19] Delworth TL, Zeng F (2014). Regional rainfall decline in Australia attributed to anthropogenic greenhouse gases and ozone levels. Nat. Geosci..

[20] Knutson TR, Zeng F (2018). Model assessment of observed precipitation trends over land regions: Detectable human influences and possible low bias in model trends. J. Clim..

[21] Timbal B, Arblaster JM, Power S (2006). Attribution of the late-twentieth-century rainfall decline in southwest Australia. J. Clim..

[22] Hope, P. *et al. Southern and South-Western Flatlands Cluster Report*. *Climate Change in Australia Projections for Australia’s Natural Resource Management Regions: Cluster Reports* (2015).

[23] Polade SD, Gershunov A, Cayan DR, Dettinger MD, Pierce DW (2017). Precipitation in a warming world: Assessing projected hydro-climate changes in California and other Mediterranean climate regions. Sci. Rep..

[24] Hope P (2015). Seasonal and regional signature of the projected southern Australian rainfall reduction. Aust. Meteorol. Oceanogr. J..

[25] Gutiérrez, J. M. *et al.* Atlas. in *Climate Change 2021: The Physical Science Basis. Contribution of Working Group I to the Sixth Assessment Report of the Intergovernmental Panel on Climate Change* (eds. Masson-Delmotte, V. *et al*.). http://interactiveatlas.ipcc.ch/ (2021).

[26] Hausfather Z, Peters GP (2020). RCP8.5 is a problematic scenario for near-term emissions. Proc. Natl. Acad. Sci. USA.

[27] Hausfather Z, Peters GP (2020). Emissions: The ‘business as usual’ story is misleading. Nature.

[28] Schwalm CR, Glendon S, Duffy PB (2020). RCP8.5 tracks cumulative CO2 emissions. Proc. Natl. Acad. Sci. USA.

[29] Masson-Delmotte, V. *et al*. Intergovernmental Panel on Climate Change. Summary for Poliymakers. in *Global Warming of 1.5°C: An IPCC Special Report on the Impacts of Global Warming of 1.5°C Above Pre-industrial Levels and Related Global Greenhouse Gas Emission Pathways, in the Context of Strengthening the Global Response to the Threat of Climate Change* (2018).

[30] Rauniyar SP, Power SB (2020). The impact of anthropogenic forcing and natural processes on past, present, and future rainfall over Victoria, Australia. J. Clim..

[31] Ziehn T (2020). The Australian earth system model: ACCESS-ESM1.5. J. South. Hemisph. Earth Syst. Sci..

[32] Grose MR (2020). Insights from CMIP6 for Australia’s future climate. Earth’s Futur..

[33] Rauniyar SP, Power SB (2023). Past and future rainfall change in sub-regions of Victoria, Australia. Clim. Change.

[34] Power S, Delage F, Wang G, Smith I, Kociuba G (2017). Apparent limitations in the ability of CMIP5 climate models to simulate recent multi-decadal change in surface temperature: Implications for global temperature projections. Clim. Dyn..

[35] Swart NC (2019). The Canadian earth system model version 5 (CanESM5.0.3). Geosci. Model Dev..

[36] Hope PK (2006). Projected future changes in synoptic systems influencing southwest Western Australia. Clim. Dyn..

[37] Kelder T (2022). Interpreting extreme climate impacts from large ensemble simulations: Are they unseen or unrealistic?. Environ. Res. Lett..

[38] Timbal B, Arblaster JM (2006). Land cover change as an additional forcing to explain the rainfall decline in the south west of Australia. Geophys. Res. Lett..

[39] Pitman AJ (2004). Impact of land cover change on the climate of southwest Western Australia. J. Geophys. Res..

[40] Hurtt GC (2020). Harmonization of global land use change and management for the period 850–2100 (LUH2) for CMIP6. Geosci. Model Dev..

[41] Lim EP, Hendon HH, Thompson DWJ (2018). Seasonal evolution of stratosphere-troposphere coupling in the southern hemisphere and implications for the predictability of surface climate. J. Geophys. Res. Atmos..

[42] McKay RC (2023). Can southern Australian rainfall decline be explained? A review of possible drivers. WIREs Clim. Chang..

[43] Grose MR, King AD (2023). The circulation and rainfall response in the southern hemisphere extra-tropics to climate stabilisation. Weather Clim. Extrem..

[44] Burls NJ, Fedorov AV (2017). Wetter subtropics in a warmer world: Contrasting past and future hydrological cycles. Proc. Natl. Acad. Sci. USA..

[45] Sniderman JMK (2019). Southern Hemisphere subtropical drying as a transient response to warming. Nat. Clim. Change.

[46] Cullen LE, Grierson PF (2009). Multi-decadal scale variability in autumn-winter rainfall in south-western Australia since 1655 AD as reconstructed from tree rings of Callitris columellaris. Clim. Dyn..

[47] Griffiths AD, Treble PC, Hope P, Rudeva I (2022). Rainfall stable water isotope variability in coastal southwestern Western Australia and its relationship to climate on multiple timescales. J. Geophys. Res. Atmos..

[48] Priestley, S., Griths, A., Baker, A., Abram, N. & Meredith, K. Caves provide early warning of unprecedented decrease in rainfall recharge of groundwater. *Commun. Earth Environ.* (2022).

[49] Breshears DD (2021). Underappreciated plant vulnerabilities to heat waves. New Phytol..

[50] Evans, A., Jones, D., Smalley, R. & Lellyett, S. *An Enhanced Gridded Rainfall Dataset scheme for Australia*. *Bureau Research Report No. 41* (2020).

[51] Jones PW (1999). First- and second-order conservative remapping schemes for grids in spherical coordinates. Mon. Weather Rev..

[52] Rauniyar SP, Protat A, Kanamori H (2017). Uncertainties in TRMM-Era multisatellite-based tropical rainfall estimates over the Maritime Continent. Earth Space Sci..

[53] Jones D, Wang W, Fawcett R (2009). High-quality spatial climate data-sets for Australia. Aust. Meteorol. Oceanogr. J..

[54] Jeffrey SJ, Carter JO, Moodie KB, Beswick AR (2001). Using spatial interpolation to construct a comprehensive archive of Australian climate data. Environ. Model. Softw..

[55] Harris I, Osborn TJ, Jones P, Lister D (2020). Version 4 of the CRU TS monthly high-resolution gridded multivariate climate dataset. Sci. Data.

[56] Taylor KE, Stouffer RJ, Meehl GA, Taylor RSGM (2012). An overview of CMIP5 and the experiment design. Bull. Am. Meteorol. Soc..

[57] Eyring V (2016). Overview of the coupled model intercomparison project phase 6 (CMIP6) experimental design and organization. Geosci. Model Dev..

[58] O’Neill BC (2016). The scenario model intercomparison project (ScenarioMIP) for CMIP6. Geosci. Model Dev..

[59] van Vuuren DP (2011). The representative concentration pathways: An overview. Clim. Change.

[60] Efron B, Tibshirani RJ (1994). An Introduction to the Bootstrap.

