# Supplementary material for: The role of internal variability and external forcing on southwestern Australian rainfall: prospects for very wet or dry years
Source: Sci Rep. 2023 Dec 7;13:21578. doi: 10.1038/s41598-023-48877-w (PMC10703814; doi:10.1038/s41598-023-48877-w)
Supplement: Supplementary file 1 — Supplementary Information. [file 41598_2023_48877_MOESM1_ESM.docx]

**Supplementary Material**

The role of internal variability and external forcing on southwestern Australian rainfall – prospects for very wet or dry years

**Surendra P. Rauniyar^1,^ *, Pandora Hope^1,2^, Scott B. Power^2,3,4^, Michael Grose^5^, David Jones^1^**

Surendra P. Rauniyar

^1^Australian Bureau of Meteorology, Melbourne, Australia

E-mail: [Surendra.rauniyar@bom.gov.au](mailto:Surendra.rauniyar@bom.gov.au)

ORCID: 0000-0001-8178-334X

Pandora Hope

^1^Australian Bureau of Meteorology, Melbourne, Australia

^2^ARC Centre of Excellence for Climate Extremes, Australia

ORCID: 0000-0002-9631-8181

Scott B. Power

^2^ARC Centre of Excellence for Climate Extremes, Australia

^3^School of Earth, Atmosphere and Environment, Monash University, Melbourne, Australia

^4^Formerly, Centre for Applied Climate Sciences, University of Southern Queensland, Queensland, Australia

ORCID: 0000-0002-9596-4368

Michael Grose

^5^CSIRO Oceans and Atmosphere, Hobart, Australia

ORCID: 0000-0001-8012-9960

David A. Jones

^1^Australian Bureau of Meteorology, Melbourne, Australia

ORCID: 0000-0001-9868-0013

**Supplementary Table S1.** CMIP5 model details. Models shown have at least 500 years of piControl simulations, historical (1900 – 2005) simulations under all (Natural plus anthropogenic) forcings, and future (2006- 2100) projections under RCP8.5 and RCP4.5 scenarios. Models shown in bold do not have future projections under a low emission (RCP2.6) scenario. Only first ensemble member (i.e., r1i1p1) is being used in this study.

| **Model Name** | **piControl** | **Historical** | **Future scenarios** | | | **Atmospheric grid**  **lat/lon resolution** |
| --- | --- | --- | --- | --- | --- | --- |
|  |  | **ALL-forcing** | **RCP8.5** | **RCP4.5** | **RCP2.6** |  |
| **ACCESS1-0** | 1 | 1 | 1 | 1 |  | 1.2 × 1.9 |
| **ACCESS1-3** | 1 | 1 | 1 | 1 |  | 1.2 × 1.9 |
| bcc-csm1-1 | 1 | 1 | 1 | 1 | 1 | 2.8 × 2.8 |
| BNU-ESM | 1 | 1 | 1 | 1 | 1 | 2.8 × 2.8 |
| CanESM2 | 1 | 1 | 1 | 1 | 1 | 2.8 × 2.8 |
| CCSM4 | 1 | 1 | 1 | 1 | 1 | 0.9 × 1.2 |
| **CESM1-BGC** | 1 | 1 | 1 | 1 |  | 0.9 × 1.2 |
| **CMCC-CMS** | 1 | 1 | 1 | 1 |  | 1.9 × 1.9 |
| CNRM-CM5 | 1 | 1 | 1 | 1 | 1 | 1.4 × 1.4 |
| CSIRO-Mk3-6-0 | 1 | 1 | 1 | 1 | 1 | 1.9 × 1.9 |
| FGOALS-g2 | 1 | 1 | 1 | 1 | 1 | 2.8 × 4.7 |
| FGOALS-s2 | 1 | 1 | 1 | 1 | 1 | 1.6 × 2.8 |
| GFDL-CM3 | 1 | 1 | 1 | 1 | 1 | 2.0 × 2.5 |
| GFDL-ESM2G | 1 | 1 | 1 | 1 | 1 | 2.0 × 2.5 |
| GFDL-ESM2M | 1 | 1 | 1 | 1 | 1 | 2.0 × 2.5 |
| HadGEM2-ES | 1 | 1 | 1 | 1 | 1 | 1.2 × 1.9 |
| **INMCM4** | 1 | 1 | 1 | 1 |  | 1.5 × 2.0 |
| IPSL-CM5A-LR | 1 | 1 | 1 | 1 | 1 | 1.9 × 3.7 |
| MIROC-ESM | 1 | 1 | 1 | 1 | 1 | 2.8 × 2.8 |
| MIROC5 | 1 | 1 | 1 | 1 | 1 | 1.4 × 1.4 |
| MPI-ESM-LR | 1 | 1 | 1 | 1 | 1 | 1.9 × 1.9 |
| MPI-ESM-MR | 1 | 1 | 1 | 1 | 1 | 1.9 × 1.9 |
| MRI-CGCM3 | 1 | 1 | 1 | 1 | 1 | 1.1 × 1.1 |
| NorESM1-M | 1 | 1 | 1 | 1 | 1 | 1.9 × 2.5 |
| **Total** | **24** | **24** | **24** | **24** | **19** |  |

**Supplementary Table S2.** CMIP6 model details. Models shown have at least 500 years of piControl simulations, historical (1900 - 2014) simulations under all (Natural plus anthropogenic) forcings, and future (2015-2100) projections under SSP5.85 scenarios. Models shown in bold have future projections missing under other scenarios. Only first ensemble member (i.e., r1i1p1f1) is being used in this study, except 40 members large-ensemble runs of ACCESS-ESM1-5 (r1-40i1p1f1).

| **Model Names** | **piControl** | **Historical** | **Future Scenarios** | | | | **Atmospheric grid lat/lon resolution** |
| --- | --- | --- | --- | --- | --- | --- | --- |
|  | **500 years** | **ALL-forcing** | **SSP5.85** | **SSP3.70** | **SSP2.45** | **SSP1.26** |  |
| ACCESS-CM2 | 1 | 1 | 1 | 1 | 1 | 1 | 1.2 × 1.8 |
| ACCESS-ESM1-5 | 1 | 1 | 1 | 1 | 1 | 1 | 1.2 × 1.8 |
| AWI-CM-1-1-MR | 1 | 1 | 1 | 1 | 1 | 1 | 0.9 × 0.9 |
| BCC-CSM2-MR | 1 | 1 | 1 | 1 | 1 | 1 | 1.1 × 1.1 |
| CanESM5 | 1 | 1 | 1 | 1 | 1 | 1 | 2.8 × 2.8 |
| **CESM2** | 1 | 1 | 1 |  |  | 1 | ~1.0 |
| **CIESM** | 1 | 1 | 1 |  | 1 | 1 | 1.0 × 1.0 |
| CMCC-CM2-SR5 | 1 | 1 | 1 | 1 | 1 | 1 | ~0.9 |
| CMCC-ESM2 | 1 | 1 | 1 | 1 | 1 | 1 | 0.9 × 1.25 |
| EC-Earth3 | 1 | 1 | 1 | 1 | 1 | 1 | 0.7 × 0.7 |
| **EC-Earth3-CC** | 1 | 1 | 1 |  | 1 |  | ~1.0 |
| EC-Earth3-Veg | 1 | 1 | 1 | 1 | 1 | 1 | 0.7 × 0.7 |
| EC-Earth3-Veg-LR | 1 | 1 | 1 | 1 | 1 | 1 | 0.7 × 0.7 |
| FGOALS-f3-L | 1 | 1 | 1 | 1 | 1 | 1 | 2.3 × 2.0 |
| FGOALS-g3 | 1 | 1 | 1 | 1 | 1 | 1 | 2.3 × 2.0 |
| **GFDL-CM4** | 1 | 1 | 1 |  | 1 |  | 1.0 × 1.3 |
| GFDL-ESM4 | 1 | 1 | 1 | 1 | 1 | 1 | 1.0 × 1.3 |
| INM-CM4-8 | 1 | 1 | 1 | 1 | 1 | 1 | 1.5 × 2.0 |
| INM-CM5-0 | 1 | 1 | 1 | 1 | 1 | 1 | 1.5 × 2.0 |
| IPSL-CM6A-LR | 1 | 1 | 1 | 1 | 1 | 1 | 1.3 × 2.5 |
| MIROC6 | 1 | 1 | 1 | 1 | 1 | 1 | 1.4 × 1.4 |
| MPI-ESM1-2-HR | 1 | 1 | 1 | 1 | 1 | 1 | ~0.9 |
| MPI-ESM1-2-LR | 1 | 1 | 1 | 1 | 1 | 1 | ~2.0 |
| MRI-ESM2-0 | 1 | 1 | 1 | 1 | 1 | 1 | 1.1 × 1.1 |
| NorESM2-MM | 1 | 1 | 1 | 1 | 1 | 1 | 0.9 × 0.9 |
| TaiESM1 | 1 | 1 | 1 | 1 | 1 | 1 | 0.9 × 0.9 |
| **Total** | **26** | **26** | **26** | **22** | **25** | **24** |  |

**Supplementary Table S3.** Summary of the percentage change in cool (May-Oct) season area-averaged rainfall over the SWWA region for different 20-year periods from 1901-1920 through to 2081-2100 using CMIP5 historical plus RCP8.5 scenarios. The changes are relative to 1901-1960 period average over the south-west Western Australia (SWWA) region. The values inside the square brackets from 2001-2020 onwards show changes for RCP4.5 (left) and RCP2.6 (right) scenarios. The multi-model mean for the first 60-year period of models with preindustrial runs is 49.2 mm month^-1^ and the standard deviation of the distribution of 20-year percentage changes in rainfall under preindustrial runs (internal variability) is 4.45% and the minimum and maximum values of the distribution are -18% and 24%, respectively.

| **Period** | **No. of Models** | **mean** | **min** | **5%** | **25%** | **50%** | **75%** | **95%** | **max** |
| --- | --- | --- | --- | --- | --- | --- | --- | --- | --- |
| 1901-1920 | 24 | 1.3 | -6.7 | -4.6 | -0.7 | 1.2 | 3.1 | 5.9 | 9.6 |
| 1921-1940 | 24 | 0.3 | -5.0 | -4.8 | -1.5 | 0.4 | 1.6 | 4.8 | 6.2 |
| 1941-1960 | 24 | -1.6 | -7.8 | -5.9 | -4.0 | -1.6 | 0.3 | 6.1 | 7.4 |
| 1961-1980 | 24 | -2.1 | -11.9 | -8.3 | -4.6 | -2.0 | 1.5 | 4.0 | 6.4 |
| 1971-2000 | 24 | -3.4 | -10.7 | -9.8 | -6.0 | -3.5 | -0.9 | 1.3 | 4.8 |
| 1981-2000 | 24 | -3.8 | -13.6 | -9.6 | -7.0 | -3.8 | -0.3 | 3.5 | 3.9 |
| 2001-2020 | 24  [24, 19] | -8.7  [-9.4, -9.5] | -18.4  [-21.7, -19.8] | -15.9  [-20.8, -19.1] | **-11.1**  **[-13.7, -12.5]** | **-8.9**  **[-7.9, -11.6]** | **-5.7**  **[-5.5, -7.4]** | -1.5  [-0.8, -0.2] | 0.2  [0.2, -0.2] |
| 2011-2030 | 24  [24, 19] | -11.8  [-12.4, -11.8] | -26.1  [-21.6, -24.9] | -20.7  [-21.0, -19.7] | -14.9  [-16.0, -16.8] | -11.2  [-13.7, -10.3] | -7.4  [-8.2, -8.2] | -4.3  [-4.8, -2.1] | 0.1  [-2.5, -1.1] |
| 2021-2040 | 24  [24, 19] | -14.8  [-14.3, -13.9] | -28.1  [-22.0, -23.5] | -22.3  [-20.6, -22.7] | -19.0  [-18.9, -18.2] | -16.6  [-16.0, -13.9] | -11.8  [-10.7, -9.7] | -3.5  [-5.2, -5.8] | 3.6  [-3.4, -3.4] |
| 2031-2050 | 24  [24, 19] | -17.5  [-16.0, -14.8] | -30.3  [-24.9, -29.0] | -25.3  [-24.1, -28.1] | -23.8  [-20.6, -18.0] | -18.5  [-17.6, -15.3] | -13.2  [-11.5, -10.5] | -1.8  [-5.4, -5.1] | 3.0  [-4.8, -2.8] |
| 2041-2060 | 24  [24, 19] | -21.5  [-18.2, -14.3] | -35.8  [-30.9, -32.3] | -31.3  [-29.4, -24.1] | -28.0  [-23.1, -17.4] | -22.9  [-19.6, -13.1] | -15.7  [-11.8, -11.3] | -9.2  [-5.2, -5.1] | -8.6  [-3.3, -4.3] |
| 2051-2070 | 24  [24, 19] | -26.5  [-19.7, -12.9] | -39.2  [-31.1, -28.7] | -37.3  [-30.9, -24.4] | -32.5  [-26.0, -18.6] | -26.9  [-20.9, -12.3] | -21.0  [-16.5, -7.6] | -12.6  [-5.3, -3.2] | -10.8  [-1.5, 3.8] |
| 2061-2080 | 24  [24, 19] | -30.0  [-19.3, -13.6] | -43.7  [-32.3, -27.3] | -43.0  [-31.5, -25.1] | -37.3  [-24.5, -18.7] | -30.1  [-19.1, -14.3] | -24.3  [-15.5, -9.0] | -16.6  [-8.5, -2.0] | -12.8  [-5.7, 2.5] |
| 2071-2090 | 24  [24, 19] | -32.6  [-19.3, -13.1] | -49.6  [-36.4, -24.9] | -46.5  [-29.6, -22.5] | -41.0  [-23.1, -16.5] | -32.8  [-19.6, -13.3] | -26.8  [-14.7, -10.0] | -16.3  [-10.0, -1.6] | -12.5  [-4.6, 1.0] |
| 2081-2100 | 24  [24, 19] | -36.5  [-20.2, -12.3] | -52.0  [-37.3, -25.8] | -50.6  [-32.6, -20.2] | -46.6  [-25.7, -17.1] | -35.8  [-20.2, -12.7] | -28.1  [-17.0, -8.7] | -21.9  [-3.6, 0.0] | -21.5  [-1.2, 0.6] |

**Supplementary Table S4.** Same as in Supplementary Table 3, but for CMIP6. The values inside the top and bottom square brackets from 2001-2020 onwards show changes for [SSP5.85, SSP3.70] and [SSP2.45, SSP1.26] scenarios. The multi-model mean for the first 60-year period of models with preindustrial runs is 49.5 mm month^-1^ and the standard deviation of the distribution of 20-year percentage changes in rainfall under preindustrial runs (internal variability) is 4.52% and the minimum and maximum values of the distribution are -18% and 23%, respectively.

| **Period** | **No. of Models** | **mean** | **min** | **5%** | **25%** | **50%** | **75%** | **95%** | **max** |
| --- | --- | --- | --- | --- | --- | --- | --- | --- | --- |
| 1901-1920 | 26 | 0.5 | -6.1 | -4.6 | -1.3 | 1.3 | 2.9 | 4.6 | 7.0 |
| 1921-1940 | 26 | -0.7 | -4.0 | -3.9 | -2.6 | -0.9 | 0.6 | 2.8 | 6.8 |
| 1941-1960 | 26 | 0.2 | -3.9 | -3.5 | -1.4 | -0.3 | 2.0 | 4.3 | 4.4 |
| 1961-1980 | 26 | -1.0 | -9.1 | -7.7 | -4.7 | -1.6 | 2.7 | 6.2 | 7.4 |
| 1971-2000 | 26 | -3.1 | -10.9 | -8.8 | -5.5 | -4.1 | -0.4 | 3.0 | 7.1 |
| 1981-2000 | 26 | -4.1 | -11.6 | -10.1 | -7.2 | -4.7 | -2.0 | 2.3 | 11.3 |
| 2001-2020 | [26, 22]  [25, 24] | [-9.0, -8.4]  [-8.5, -9.0] | [-17.6, -16.3]  [-17.1, -17.4] | [-16.1, -13.9]  [-16.2, -16.6] | **[-12.3, -12.8]**  **[-11.9, -13.3]** | **[-8.6, -8.9]**  **[-8.3, -9.5]** | **[-6.8, -6.2]**  **[-5.6, -5.8]** | [-1.2, 1.0]  [0.3, -0.7] | [0.3, 0.3]  [1.3, 2.6] |
| 2011-2030 | [26, 22]  [25, 24] | [-12.4, -11.0]  [-11.2, -11.3] | [-22.2, -18.1]  [-19.3, -20.1] | [-21.0, -16.1]  [-18.9, -18.3] | [-15.5, -12.9]  [-13.9, -15.6] | [-12.0, -11.6]  [-11.0, -11.0] | [-9.4, -10.0]  [-8.6, -7.6] | [-3.9, -2.0]  [-2.6, -0.7] | [-3.1, -3.1]  [2.4, 1.7] |
| 2021-2040 | [26, 22]  [25, 24] | [-14.3, -13.5]  [-13.5, -12.6] | [-24.7, -20.8]  [-24.9, -20.6] | [-23.4, -19.9]  [-20.5, -20.3] | [-15.8, -16.1]  [-18.1, -15.6] | [-14.5, -13.5]  [-14.3, -12.3] | [-11.4, -11.4]  [-10.4, -9.4] | [-6.5, -5.9]  [-5.3, -5.4] | [-5.3, -5.3]  [-0.4, -3.3] |
| 2031-2050 | [26, 22]  [25, 24] | [-16.9, -15.9]  [-15.9, -14.7] | [-29.0, -24.4]  [-27.9, -23.8] | [-26.0, -23.3]  [-25.7, -22.8] | [-19.9, -19.2]  [-18.5, -18.9] | [-17.7, -16.2]  [-16.1, -14.3] | [-12.7, -13.5]  [-12.2, -11.2] | [-7.5, -9.7]  [-9.6, -7.3] | [-5.5, -5.5]  [-3.1, -4.8] |
| 2041-2060 | [26, 22]  [25, 24] | [-19.5, -18.7]  [-17.7, -15.5] | [-33.3, -28.3]  [-28.0, -24.9] | [-27.5, -25.9]  [-26.7, -23.9] | [-23.1, -21.5]  [-21.2, -20.7] | [-19.8, -18.8]  [-17.5, -15.1] | [-16.8, -15.3]  [-14.3, -10.5] | [-9.0, -11.1]  [-9.4, -6.4] | [-6.0, -6.0]  [-5.3, -5.7] |
| 2051-2070 | [26, 22]  [25, 24] | [-23.3, -21.3]  [-19.4, -14.4] | [-35.6, -32.1]  [-27.6, -23.5] | [-32.7, -31.4]  [-27.0, -22.6] | [-28.5, -24.3]  [-23.9, -17.3] | [-22.4, -21.8]  [-19.5, -14.1] | [-20.6, -18.5]  [-15.9, -11.5] | [-14.3, -10.7]  [-8.6, -6.1] | [-11.7, -11.7]  [-6.6, -4.4] |
| 2061-2080 | [26, 22]  [25, 24] | [-28.4, -23.5]  [-20.9, -14.6] | [-40.6, -33.7]  [-37.1, -23.7] | [-37.8, -33.2]  [-31.4, -21.6] | [-32.6, -28.0]  [-23.5, -18.2] | [-28.8, -23.9]  [-20.4, -15.1] | [-22.3, -20.3]  [-17.5, -10.6] | [-20.3, -10.8]  [-11.8, -8.1] | [-15.1, -15.1]  [-9.7, -3.4] |
| 2071-2090 | [26, 22]  [25, 24] | [-32.6, -26.7]  [-21.1, -14.9] | [-46.8, -40.2]  [-30.3, -26.8] | [-44.6, -35.8]  [-28.6, -24.3] | [-37.7, -31.7]  [-26.1, -16.5] | [-32.3, -26.5]  [-21.5, -15.3] | [-27.2, -23.2]  [-16.3, -11.2] | [-23.2, -14.3]  [-12.1, -7.1] | [-13.3, -13.3]  [-10.8, -5.2] |
| 2081-2100 | [26, 22]  [25, 24] | [-35.2, -29.7]  [-21.2, -13.2] | [-50.0, -45.0]  [-31.4, -25.2] | [-48.1, -40.6]  [-30.0, -21.2] | [-42.3, -33.5]  [-25.5, -17.4] | [-33.9, -30.1]  [-21.1, -12.4] | [-31.2, -26.8]  [-19.1, -10.5] | [-24.7, -19.2]  [-12.4, -5.2] | [-8.1, -8.1]  [-7.0, -0.7] |


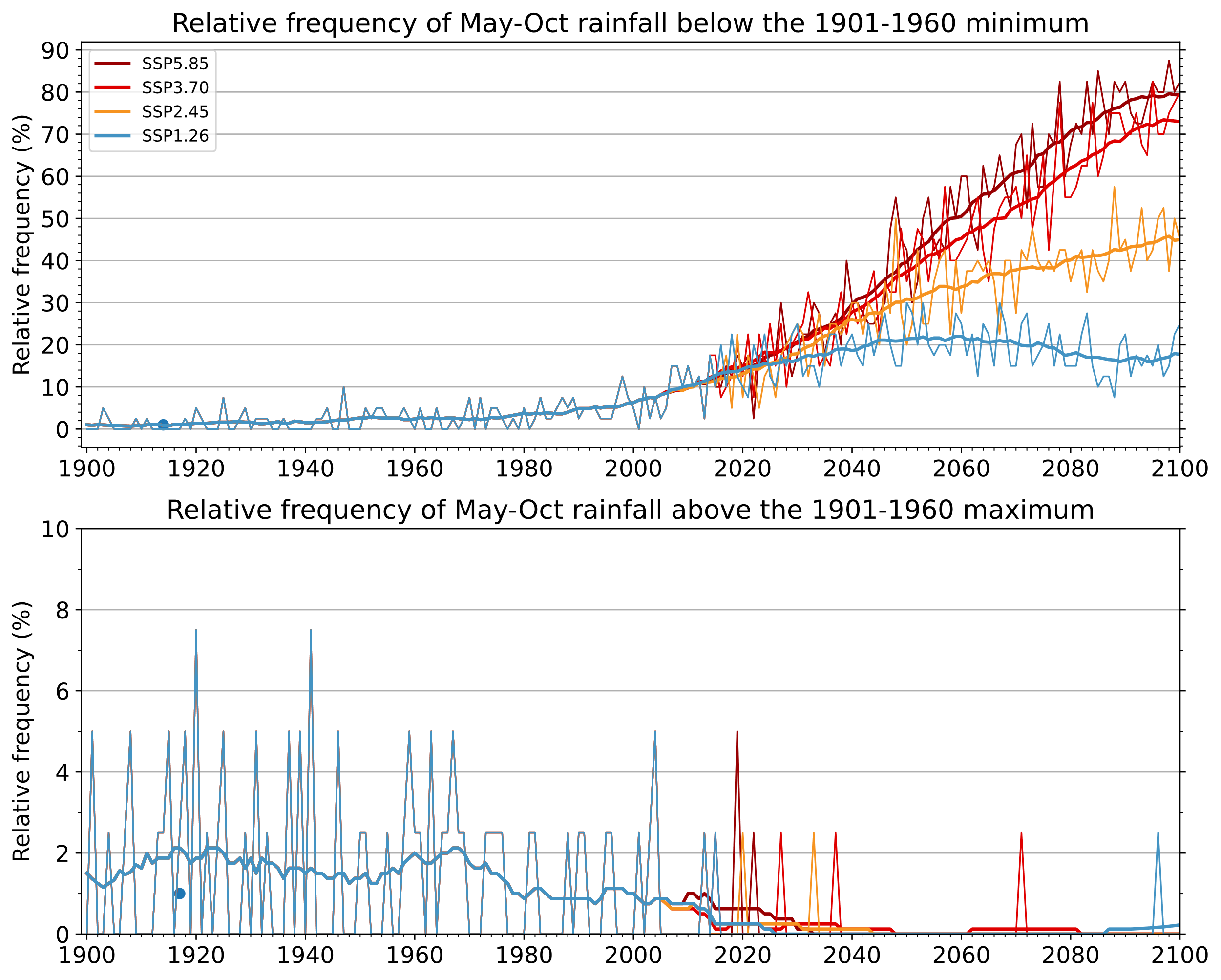


**Supplementary Figure S1.** The relative frequency of the ACCESS-ESM1.5 40 ensemble members that show rainfall (top) below the minimum and (bottom) above the maximum rainfall of their 1901-1960 period under historical and four different SSPs as shown in the legend.
